# The association of admission ionized calcium with outcomes of thrombolysed patients with anterior circulation ischemic stroke

**DOI:** 10.1002/brb3.2844

**Published:** 2022-12-07

**Authors:** Yan‐Li Qi, Qiong Wu, Xiao‐Qiu Li, Zhong‐He Zhou, Cheng Xia, Xin‐Hong Wang, Hui‐Sheng Chen

**Affiliations:** ^1^ Department of Neurology General Hospital of Northern Theater Command Shenyang People's Republic of China

**Keywords:** acute ischemic stroke, ionized calcium, prognosis, thrombolysis

## Abstract

**Background and purpose:**

The relationship between ionized calcium and prognosis of ischemic stroke is controversial. We aim to determine the relationship of admission ionized calcium levels with acute ischemic stroke (AIS) after intravenous thrombolysis (IVT).

**Methods:**

Consecutive anterior circulation AIS patients treated with recombinant tissue plasminogen activator (rt‐PA) were retrospectively enrolled. According to ionized calcium quartiles, the patients were divided into four groups and clinical data were analyzed between groups. Ionized calcium was entered into logistic regression analysis in two models, separately: model 1, calcium as a continuous variable (per 1‐mmol/L increase), and model 2, calcium as the four‐categorized variable (being collapsed into quartiles: Q1–Q4). Early neurologic improvement (ENI) was defined as improvement of four or more points at 24 h after intravenous rt‐PA, while long‐term good outcome as the modified Rankin Scale (mRS) 0–1 at 90 days.

**Results:**

A total of 546 patients met the study criteria (mean age was 63.51 ± 11.26 years and 365 [66.8%] were men). The median admission National Institute of Health Stroke Scale was 9 (range 4 to 15). When not adjusted, in model 1: ionized calcium was related to good outcome (odds ratio [OR] 69.061, 95%CI: 1.638–2911.111, *p*=0.027), but not ENI (OR 14.097, 95%CI: 0.133–1492.596, *p*=0.266); in model 2: compared with Q4, while good outcome was less common in Q1 (OR 0.623, 95%CI: 0.388–0.999, *p*=0.049). After adjusting for confounding factors, calcium in Q2 (OR 0.502, 95%CI: 0.253–0.997, *p*=0.049) was independently associated with ENI, but no matter as a continuous variable or categorized variable, ionized calcium displayed no association with a good outcome.

**Conclusion:**

The current results found that ionized calcium might be associated with early neurological improvement, but had no association with 3 months' outcome in anterior circulation AIS patients after IVT.

## Graphical abstract

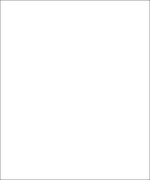

## 1 INTRODUCTION

Previous studies have investigated the relationship of calcium with clinical outcome in ischemic stroke patients, but the results are controversial. For example, most studies supported its association with outcome (Buck et al., 2007; Chung et al., 2015; D'Erasmo et al., 1998; Ovbiagele et al., 2006) while one study denied the association (Ovbiagele et al., 2008). The controversy is not clear. One possible cause is that the difference may be explained by the physiologic characteristics of Ca^2+^. Calcium in the serum exists in three fractions: 50% is a biologically active ionized state, 40% is bound to the serum proteins (mainly albumin), and 10% is conjugated to anions such as bicarbonate and citrate (Bushinsky & Monk, 1998). Of those, ionized Ca^2+^ is the physiologically active component of serum Ca^2+^ levels. Thus, it is important that ionized Ca^2+^ should be used to investigate its association with clinical outcomes after ischemic stroke. Intravenous thrombolysis (IVT) within 4.5 h from symptom onset is a well approved and standard treatment for acute ischemic stroke (AIS). Many factors such as older age, diabetes mellitus, a history of atrial fibrillation (AF), dehydration, and initial stroke severity were found to be related to the poor outcome of thrombolysed patients (Li et al., 2017; Stefanovic Budimkic et al., 2017). To our best knowledge, no study has determined the role of calcium in the prognosis of AIS after IVT. In this context, the current study aimed to investigate the association between ionized calcium and early and long‐term prognosis of thrombolysed patients with AIS.

## 2 MATERIALS AND METHODS

### 2.1 Subjects

From our prospective thrombolysis database (Wang et al., 2021), consecutive patients receiving IVT were recruited in our department from July 2012 to July 2019. The inclusion criteria were: (1) age ≥18 years; (2) diagnosis of acute anterior circulation AIS; (3) treatment with intravenous recombinant tissue plasminogen activator (rt‐PA) (alteplase 0.9 mg/kg, up to a maximum of 90 mg/kg) within 4.5 h of symptom onset; (4) ionized calcium level collected on admission; and (5) computed tomography (CT) performed on admission and 24 h after IVT. Patients were excluded if: (1) posterior circulation stroke; (2) they received endovascular intervention; (3) incomplete clinical data. The study was approved by the Ethics Committee of the Institutional Review Board of General Hospital of Northern Theater Command (the former General Hospital of Shen Yang Military Region) (IRB: k2016‐11) and the board waived the need for patient consent due to the retrospective nature.

### 2.2 Data collection

The collected data include demographics (age and gender), risk factors (hypertension, diabetes mellitus, coronary artery disease, AF, stroke, alcohol consumption and smoking), clinical data on admission (systolic pressure, diastolic pressure, National Institutes of Health Stroke Scale [NIHSS] score), and laboratory data (ionized calcium, hematological and biochemical laboratory tests, and so on). Ionized calcium was measured on admission by an arterial blood gas analyzer (normal reference range: 1.10–1.34 mmol/L), and patients were divided into four quartiles (Q1–Q4) according to ionized calcium level. Hypertension, diabetes mellitus, coronary artery disease, or AF was determined if a definite history existed or the condition was definitely diagnosed at discharge. Symptomatic intracranial hemorrhage was defined as exacerbation of clinical symptoms with an increase of ≥ 4 points in NIHSS score caused by intracranial hemorrhage (Larrue et al., 2001). Etiological classification of patients was done according to Trial of Org 10172 in Acute Stroke Treatment (TOAST) criteria (Adams et al., 1993). Modified Rankin Scale (mRS) scores at 90 days were recorded.

### 2.3 Outcome assessments

All patients were assessed at 3 months using mRS. A good outcome was defined as functional independence (an mRS score of 0 or 1). A score of 2 or greater on the mRS was used to define a poor outcome. Early neurologic improvement (ENI) was defined as improvement of four or more points at 24 h after intravenous rt‐PA (Nagaraja et al., 2014).

### 2.4 Statistical analysis

All data analysis was performed by SPSS 21.0. Continuous variables were shown as mean ± standard deviation or the median (interquartiles), and categorical variables were shown as the number of cases and percentage. As the significance of the differences in terms of mean values was assessed by one‐way variance analysis, the significance of the differences of the median values was analyzed by the Kruskal–Wallis test. Categorical variables were evaluated with Pearson chi‐square or Fisher's exact chi‐square test results. Univariate and multivariate logistic regression analyses were used to calculate the relationship between ionized calcium and adverse prognosis. For multivariate analysis, the pool of covariates was determined based on univariate analysis at the *p*<0.1 level and previous literature (Guo et al., 2015). The results were reported as an odds ratio (OR) and 95% confidence interval (CI). Two‐sided values of *p*<0.05 were considered statistically significant.

## 3 RESULTS

**FIGURE 1:**
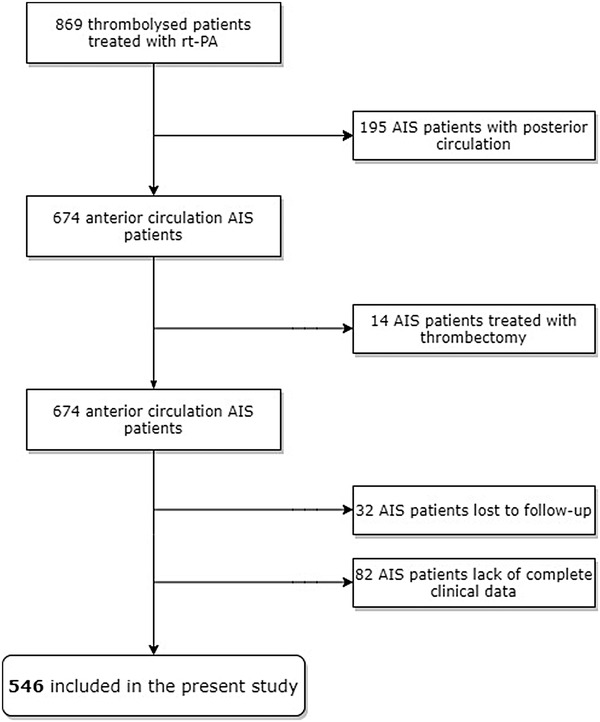
The flowchart of patient enrollment

**FIGURE 2:**
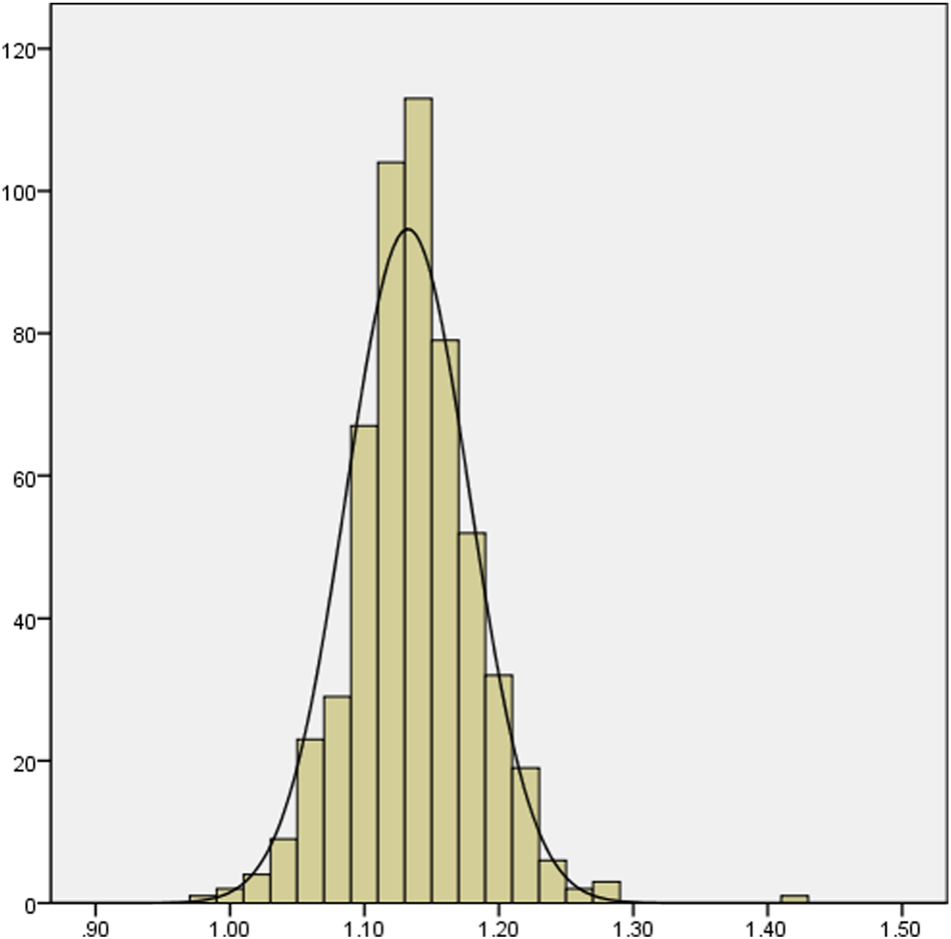
The distribution of calcium in our cohort

From July 2012 to July 2019, 869 thrombolysed patients with AIS were enrolled in the present study. A total of 323 patients were excluded due to different reasons: 195 patients with posterior circulation, 14 patients treated with thrombectomy, and 114 patients with lack of complete clinical data (Figure 1). Finally, 546 patients met the study criteria. As shown in Figure 2, ionized calcium was normal distribution in our cohort. Table 1 shows the baseline characteristics of all patients according to ionized calcium quartiles. The mean age was 63.51 ± 11.26 years, 66.8% were men, and median admission NIHSS was 9 (range 4 to 15). A total of 100 patients (18.3%) had ENI, while 252 patients (46.2%) had a good outcome (mRS 0–1) at 3 months. Table 2 shows baseline characteristics of thrombolysed patients according to ENI and 90‐day good outcome. Compared with mRS 0–1 patients, the patients with mRS 2–6 had older age, higher admission NIHSS score, lower ionized calcium at admission, higher albumin at admission, higher urea, longer onset to treatment time (OTT), more AF history, more gastrointestinal bleeding, and more sympomatic intracranial hemorrhage (sICH). TOAST subtype also showed a significant difference between two groups (Table 2).

**TABLE 1:** Baseline characteristics of patients according to ionized calcium quartiles

	Total	Ca < 1.11 mmol/L (*n* = 135)	1.11 ≤ Ca < 1.13 mmol/L (*n* = 104)	1.13 ≤ Ca < 1.16 mmol/L (*n* = 160)	Ca ≥ 1.16 mmol/L (*n* = 147)	^#^ *p*
Male (%)	365 (66.8)	90 (66.7)	67 (64.4)	112 (70.0)	96 (65.3)	.764
Age, years	63.51 ± 11.26	64.07 ± 12.18	61.85 ± 10.25	63.65 ± 11.39	64.01 ± 10.92	.405
Hypertension (%)	325 (59.9)	91 (67.4)	65 (62.5)	95 (59.4)	74 (50.3)	.029
Diabetes (%)	109 (20.0)	24 (17.8)	15 (14.4)	31 (19.4)	39 (26.5)	.094
CAD (%)	91 (16.7)	23 (17.0)	21 (20.2)	24 (15.0)	23 (15.6)	.712
AF (%)	127 (23.3)	41 (30.4)	23 (22.1)	32 (20.0)	31 (21.1)	.155
Previous stroke (%)	104 (19.0)	24 (17.8)	22 (21.2)	31 (19.4)	27 (18.4)	.920
Smoking (%)	124 (22.7)	37 (27.4)	29 (27.9)	38 (23.8)	20 (136)	.016
Drinking (%)	88 (16.1)	22 (16.3)	20 (19.2)	29 (18.1)	17 (11.6)	.323
Baseline NIHSS	9 (4, 15)	11 (4, 16)	8 (4, 13)	8 (4, 14)	8 (3, 13)	.138
SBP (mmHg)	156.30 ± 20.63	155.06 ± 22.34	157.05 ± 19.70	156.00 ± 20.38	154.47 ± 19.95	.512
DBP (mmHg)	88.35 ± 12.93	89.04 ± 13.87	89.59 ± 12.31	88.23 ± 13.57	85.89 ± 11.46	.060
Albumin (mmol/L)	38.38 ± 3.87	38.63 ± 4.51	38.33 ± 3.67	38.56 ± 3.71	37.99 ± 3.52	.506
Urea, mmol/L	6.08 ± 1.96	6.04 ± 1.89	5.63 ± 1.83	6.08 ± 1.86	6.43 ± 2.16	.017
Creatinine, mmol/L	72.36 ± 28.01	70.08 ± 20.52	71.67 ± 19.24	71.57 ± 19.28	75.79 ± 43.08	.349
OTT (hour)	3 (2.2, 3.5)	3 (2.5, 4)	3 (2, 3.5)	3 (2.2, 3.5)	2.9 (2, 3.5)	.272
sICH (%)	20 (3.7)	4 (3.0)	5 (4.8)	10 (6.3)	1 (0.7)	.063
Gastrointestinal bleeding (%)	39 (7.1)	16 (11.9)	8 (7.7)	9 (5.6)	6 (4.1)	.066
TOAST subtype						.448
LAA (%)	213 (39.0)	43 (31.9)	43 (41.3)	70 (43.8)	57 (38.8)	
SAO (%)	94 (17.2)	21 (15.6)	16 (15.4)	28 (117.5)	29 (19.7)	
CE (%)	67 (12.3)	23 (17.0)	13 (12.5)	17 (10.6)	14 (9.5)	
SUE (%)	172 (31.5)	48 (35.6)	32 (30.8)	45 (28.1)	46 (32.0)	
ENI (%)	100 (18.3)	22 (16.3)	14 (13.5)	30 (18.8)	34 (23.1)	.231
mRS ≤ 1 (%)	252 (46.2)	54 (40)	41 (39.4)	81 (50.6)	76 (51.7)	.07

Abbreviations: CAD, coronary artery disease; AF, atrial fibrillation; SBP, systolic blood pressure; DBP, diastolic blood pressure; OTT, onset to treatment; sICH, symptomatic intracranial hemorrhage; LAA, large‐artery atherosclerosis; SAO, small‐artery occlusion; CE, cardioembolic; SUE, stroke of undetermined etiology; mRS, modified Rankin Scale.

^#^ *p* for the comparison among four calcium quartiles.

**TABLE 2:** Baseline characteristics of thrombolysed patients according to early neurologic improvement and 90‐day prognosis

	ENI (+) (*n* = 100)	ENI (‐) (*n* = 446)	*p*	Good outcome (*n* = 252)	Poor outcome (*n* = 294)	*p*
Male (%)	62 (62.0)	303 (67.9)	.254	175 (69.4)	190 (64.6)	.233
Age, years	65.45 ± 9.97	63.11 ± 11.53	.066	62.25 ± 11.97	64.58 ± 10.51	.016
Hypertension (%)	53 (53.0)	272 (61.0)	.141	146 (57.9)	179 (60.9)	.484
Diabetes (%)	14 (14.0)	95 (21.3)	.099	43 (17.1)	66 (22.4)	.117
CAD (%)	18 (18.0)	73 (16.4)	.692	41 (16.3)	50 (17.0)	.818
AF (%)	17 (17.0)	110 (24.7)	.101	34 (13.5)	93 (31.6)	.000
Previous stroke (%)	16 (16.0)	88 (19.7)	.390	46 (18.3)	58 (19.7)	.662
Smoking (%)	20 (20.0)	104 (23.3)	.474	64 (25.4)	60 (20.4)	.165
Drinking (%)	16 (16.0)	72 (16.1)	.972	40 (15.9)	48 (16.3)	.886
Admission ionized calcium	1.136 ± 0.046	1.131 ± 0.046	.599	1.137 ± 0.044	1.128 ± 0.047	.025
Admission NIHSS	11 (7, 15)	8 (3, 14)	.000	4 (2, 8)	12.5 (8, 17)	.000
Admission SBP, mmHg	155.46 ± 20.22	156.24 ± 20.75	.942	156.56 ± 20.07	156.07 ± 21.13	.780
Admission DBP, mmHg	87.11 ± 11.49	88.59 ± 13.28	.191	88.44 ± 12.96	88.26 ± 12.92	.874
Admission Albumin, mmol/L	37.70 (36.00, 39.70)	38.30 (36.15, 40.80)	.111	37.95 ± 3.48	38.73 ± 4.13	.019
Urea, mmol/L	5.69 (4.98, 6.55)	5.83 (4.80, 7.13)	.444	5.85 ± 1.83	6.27 ± 2.05	.013
Creatinine, mmol/L	70.45 ± 19.84	72.91 ± 29.66	.646	72.87 ± 21.98	71.92 ± 32.30	.694
OTT, hour	2.87 ± 0.83	2.89 ± 0.91	.065	2.78 ± 0.90	2.99 ± 0.88	.006
TOAST subtype			.857			.000
LAA (%)	38 (38.0)	175 (39.2)		76 (30.2)	137 (46.6)	
SAO (%)	15 (15.0)	79 (17.7)		70 (27.8)	24 (8.2)	
CE (%)	14 (14.0)	53 (11.9)		24 (9.5)	43 (14.6)	
SUE (%)	33 (33.0)	139 (31.2)		82 (32.5)	90 (30.6)	
GB (%)	4 (4.0)	35 (7.8)	.177	4 (1.6)	35 (11.9)	.000
sICH (%)	1 (1.0)	19 (4.3)	.147	0 (0.0)	20 (6.8)	.000

Abbreviations: ENI, early neurologic improvement; CAD, coronary artery disease; AF, atrial fibrillation; NIHSS, National Institute of Health stroke scale; SBP, systolic blood pressure; DBP, diastolic blood pressure; OTT, onset to treatment; LAA, large‐artery atherosclerosis; SAO, small‐artery occlusion; CE, cardioembolic; SUE, stroke of undetermined etiology; GB, gastrointestinal bleeding; sICH, symptomatic intracranial hemorrhage.

Table 3 shows logistic regression analyses for the association of ionized calcium level at admission with ENI and 3‐month clinical outcome. For unadjusted analysis, calcium as a continuous variable displayed a significant association with good outcome (OR 69.061, 95%CI: 1.638–2911.111, *p*=0.027), and calcium as a categorical variable showed that poor outcome is more common in Q1 (OR 0.623, 95%CI: 0.388‐0.999, *p* = .049), compared with Q4, but had no association with ENI. Multivariable analysis showed that ionized calcium, as either continuous or four categorized variables, was not associated with 90‐day outcome, but the calcium in Q2 was independently associated with ENI (OR 0.502, 95%CI: 0.253‐0.997, *p* = .049), after adjusting for age, admission NIHSS, AF, albumin, urea, TOAST subtypes, gastrointestinal bleeding, sICH, and OTT.

**TABLE 3:** Association between ionize calcium and prognosis in acute ischemic stroke after thrombolysis

		ENI				mRS 0–1 at 90 days			
		Unadjusted OR 95% CI	*p*	Adjusted OR 95% CI	*p*	Unadjusted OR 95% CI	*p*	Adjusted OR 95% CI	*p*
** Model 1**	Per 1‐mmol/L	14.097 (0.133–1492.596)	0.266	28.022 (0.257–3053.200)	0.164	69.061 (1.638–2911.111)	0.027	12.302 (0.118–1277.480)	0.289
Model 2	Q1	0.647 (0.356–1.175)	0.152	0.596 (0.326–1.089)	0.093	0.**623** (0.388–0.999)	0.049	0.753 (0.414–1.371)	0.353
	Q2	0.517 (0.262–1.022)	0.058	0.502 (0.253–0.997)	0.049	0.608 (0.365–1.012)	0.055	0.566 (0.300–1.067)	0.078
	Q3	0.767 (0.442–1.332)	0.346	0.736 (0.421–1.285)	0.281	0.958 (0.612–1.499)	0.851	1.219 (0.691–2.151)	0.494
	Q4	1		1		1		1	

*Note*: Model 1, calcium included into analysis as continuous variable (per 1‐mmol/L increase); Model 2, calcium included into analysis as four‐categorized variable (stratified by quartiles).

Abbreviations: ENI, early neurological improvement; mRS, modified Rankin Scale.

Adjusted by age, admission NIHSS, atrial fibrillation, albumin, urea, TOAST subtypes, gastrointestinal bleeding, symptomatic intracranial hemorrhage, and onset to treatment.

## 4 DISCUSSION

To our best knowledge, this is the first study to investigate the relationship between admission ionized calcium levels and early and long‐term outcome of AIS patients after IVT. For the early outcome, ionized calcium, as either continuous variable, was not associated with ENI, but the calcium in Q2 was independently associated with ENI. For long‐term outcome, univariate analysis showed that ionized calcium displayed a significant association with 3‐month outcome, and poor outcome is more common in low calcium level, but the multivariable analysis did not show the association. Collectively, the results did not find the association of ionized calcium levels with 3‐month functional outcome in this population.

AIS has high fatality, disability, and recurrence rates in China (Tu et al., 2021), and IVT is currently one of the effective treatments. A lot of prognostic factors have been investigated. In recent years, several studies had investigated the prognostic significance of serum calcium levels in acute stroke patients (Guven et al., 2011; Ishfaq et al., 2017; Ovbiagele et al., 2006, 2008). In agreement with our study, one study reported that total serum Ca^2+^ levels in the early stages of AIS (< 4.5 h) was not associated with long‐term outcome (Ovbiagele et al., 2008). However, some studies reported their relationship: (1) low level of serum calcium was obviously associated with poor outcome (Chung et al., 2015; D'Erasmo et al., 1998) and more severe neurological deficit (Guven et al., 2011; Ishfaq et al., 2017; Ovbiagele et al., 2006), and bigger infarction volume (Borah et al., 2016; Buck et al., 2007) of ischemic stroke patients; (2) high levels of albumin‐corrected calcium, but not serum calcium are associated with a poor discharge outcome and a higher incidence of mortality after AIS (Chung et al., 2015); (3) an increased risk of poor functional outcome was found in the third quartile group and an increased risk of all‐cause mortality in the top quartile group of serum albumin corrected calcium levels within 24 h of hospital admission in patients with AIS and transient ischemic attack (Zhang et al., 2021).

We argue several possible explanations for the difference. First, the studied population is different: thrombolysed patients in the present study versus non‐thrombolysed patients in other studies. The explanation is also possibly supported by animal studies that venous ionized calcium is a predictor of infarct volume in the permanent, but not transient MCAO model, because the magnitude of the changes of venous ionized calcium may be masked by the benefit of reperfusion. Second, the used calcium index is different: ionized calcium in the present study versus serum calcium in other studies, while the ionized state is biologically active. Several studies have investigated the relationship of venous and arterial blood gas parameters in critically ill human patients (Ak et al., 2006; Awasthi et al., 2013; Esmaeilivand et al., 2017; Kelly et al., 2001; Khan et al., 2010; Treger et al., 2010) and in rodent models (Schwarzkopf et al., 2013; Son et al., 2010) demonstrating the good correlations between them. Last, the onset time of patients is different: less than 4.5 h in the present study versus more than 4.5 h in other studies. The explanation is also supported by the study that calcium levels were not associated with long‐term outcome in AIS patients with onset time < 4.5 h (Chung et al., 2015).

The mechanisms underlying the association of calcium with clinical outcome of stroke are not well established. It is well known that increased intracellular Ca^2+^ concentration plays an essential role in the pathological process following cerebral ischemia‐reperfusion injury (Kaushal & Schlichter, 2008). Multiple mechanisms caused intracellular calcium overload during cerebral ischemia and hypoxia, which will result in irreversible cellular injury (Choi, 1987; Grotta et al., 1986; Mattson & Mark, 1996; Siesjã, 1992). In addition, a shift of Ca^2+^ in platelets results in their activation and aggregation of microthrombus in the progression of cerebral ischemia (Leung & Nachman, 1986; Tsai et al., 2009). In vascular smooth muscle cells, Ca^2+^ influx leads to vessel constriction (Leung & Nachman, 1986) and delays reperfusion that especially follows ischemia (Akopov et al., 1994; Johansson et al., 2015). However, it is uncertain whether the intracellular influx of Ca^2+^ leads to a decrease in serum calcium levels, which is indirectly associated with outcome in AIS.

The strength of this study is the first report to investigate the association of ionized calcium with a prognosis of thrombolysed stroke patients with a relatively big sample. Based on the current finding, the relationship between long‐term drinking of milk or taking calcium drugs and the prognosis of thrombolysed stroke patients also warrants to be investigated. However, several limitations must be acknowledged. First, it is limited by the single‐center, hospital‐based, retrospective design. Second, our study only used ionized calcium at admission, but lacked data about dynamic changes of calcium. Finally, there lacked non‐thrombolysed patients as a control. Further prospective, multi‐center studies based on a large population are needed.

In conclusion, the current results found that ionized calcium might be associated with early neurological improvement, but not 3 months' clinical outcome in anterior circulation AIS patients after IVT.

## AUTHOR CONTRIBUTIONS

Hui‐Sheng Chen designed the study and revised the manuscript. Yan‐Li Qi and Xiao‐Qiu Li drafted the manuscript. Qiong Wu, Zhong‐He Zhou, Cheng Xia, and Xin‐Hong Wang collected the data. All authors read and approved the final manuscript.

## CONFLICT OF INTEREST

The authors declare that they have no conflict of interest.

### PEER REVIEW

The peer review history for this article is available at: https://publons.com/publon/10.1002/brb3.2844

## Data Availability

The datasets generated and/or analyzed during the current study are not publicly available due to the conditions of our ethics approval, but are available from the corresponding author on reasonable request.
